# Hearing Impairment and Incident Dementia: Findings from the English Longitudinal Study of Ageing

**DOI:** 10.1111/jgs.14986

**Published:** 2017-07-22

**Authors:** Hilary R. Davies, Dorina Cadar, Annie Herbert, Martin Orrell, Andrew Steptoe

**Affiliations:** ^1^ Institute of Epidemiology and Public Health University College London London United Kingdom; ^2^ Institute of Mental Health University of Nottingham Nottingham United Kingdom

**Keywords:** hearing loss, dementia, aging, epidemiology

## Abstract

**Objectives:**

To determine whether hearing loss is associated with incident physician‐diagnosed dementia in a representative sample.

**Design:**

Retrospective cohort study.

**Setting:**

English Longitudinal Study of Ageing.

**Participants:**

Adults aged 50 and older.

**Measurements:**

Cross‐sectional associations between self‐reported (n = 7,865) and objective hearing measures (n = 6,902) and dementia were examined using multinomial‐logistic regression. The longitudinal association between self‐reported hearing at Wave 2 (2004/05) and cumulative physician‐diagnosed dementia up to Wave 7 (2014/15) was modelled using Cox proportional hazards regression.

**Results:**

After adjustment for potential confounders, in cross‐sectional analysis, participants who had self‐reported or objective moderate and poor hearing were more likely to have a dementia diagnosis than those with normal hearing (self‐reported: odds ratio OR = 1.6, 95% CI = 1.1–2.4 moderate hearing; OR = 2.6, 95% CI = 1.7–3.9 poor hearing, objective: OR = 1.6, 95% CI = 1.0–2.8 moderate hearing; OR = 4.4, 95% CI = 1.9–9.9 poor hearing). Longitudinally, the hazard of developing dementia was 1.4 (95% CI = 1.0–1.9) times as high in individuals who reported moderate hearing and 1.6 (95% CI = 1.1–2.0) times as high in those who reported poor hearing.

**Conclusion:**

Older adults with hearing loss are at greater risk of dementia than those with normal hearing. These findings are consistent with the rationale that correction of hearing loss could help delay the onset of dementia, or that hearing loss itself could serve as a risk indicator for cognitive decline.

The global estimate of the number of individuals living with dementia was 46.8 million in 2015, with approximately 800,000 residing in the United Kingdom and 676,000 living in England.^1^ The estimated economic cost of dementia in the United Kingdom is approximately £23 billion per annum, which is predicted to triple by 2040.^1^ In addition, the overwhelming social impact on individuals with dementia and their families has contributed to dementia becoming a public health priority.^2^, ^3^, ^4^

A number of modifiable risk factors for dementia have been identified, including social interactions, physical activity, and type 2 diabetes mellitus.^5^ There is also evidence that hearing loss could be a risk factor.^6^, ^7^, ^8^ As with dementia, the risk of hearing loss increases with age. It was estimated that more than 3 million adults aged 50 and older in the United Kingdom had hearing loss in 2011, despite the fact that reporting or diagnosing these conditions is challenging.^9^ The Health Survey for England found that only 26% of individuals with moderate or severe objective hearing loss had previously had a formal hearing test and that 60% of individuals aged 55 and older who could have better hearing with a hearing aid had never used one.^10^

Previous longitudinal epidemiological studies conducted in the United States and Wales have provided evidence that hearing loss is independently associated with dementia,^6^, ^7^, ^11^, ^12^ but these studies have focused on adults aged 70 and older,^11^ included only men in their analysis,^6^ not included the use of hearing aids as a confounding factor,^6^, ^12^ had small sample sizes,^7^ or had no objective hearing measure.^12^ The current study was therefore designed to investigate whether subjective and objective measures of hearing loss were independently associated with dementia using cross‐sectional and longitudinal analysis in a representative sample of adults aged 50 and older in England.

## Methods

### Study Population

Data were used from a cohort of men and women aged 50 and older from the English Longitudinal Study of Ageing (ELSA),^13^ a panel study set up in 2002 with a parallel study design to the Health and Retirement Study in the United States.^13^ Face‐to‐face interviews and tests have been conducted at 2‐year intervals (Waves 1–7, 2002/03–2014/15) to obtain information on socioeconomic circumstances, physical and mental health, and cognitive function in adults as they progress into retirement.

### Outcome Measures

A three‐way assessment protocol was used to define dementia as described previously.^14^ The primary criterion was a physician diagnosis of dementia as reported by participants or informants in Waves 2 to 7.^14^ Caregivers then completed an adapted short‐form Informant Questionnaire on Cognitive Decline in the Elderly (IQCODE) for individuals who were not able to respond themselves.^14^ Caregivers were asked to compare the present functional performance of the participant 2 years before, instead of the 10‐year interval in the standard measure.^15^ Consistent with previous work, those with a score greater than 3.5 were defined as having dementia because the IQCODE has high specificity (0.84) and sensitivity (0.82) at this cut‐off.^14^, ^16^ Individuals receiving prescriptions for anticholinesterase inhibitors, N‐methyl‐D‐aspartate receptor antagonists, or other relevant medications (galantamine, rivastigmine, memantine, donepezil, or tacrine) were also determined to have dementia.^17^, ^18^ Many people with dementia do not have a formal diagnosis.^19^ These analyses should therefore be regarded as a measure of physician‐diagnosed dementia and not complete incident dementia.

### Exposure Measures

#### Self‐Reported Hearing

Participants were asked to rate their hearing from 1 (excellent) to 5 (poor) in Waves 1 to 7.^20^ Individuals with hearing aids were asked to rate their hearing based on when they used their hearing aid.^20^ Self‐reported hearing was used at Wave 2 (2004/05, longitudinal analysis) and Wave 7 (2014/15, cross‐sectional analysis). There were originally five self‐reported hearing groups (excellent, very good, good, fair, poor); fair and poor (poor) and excellent and very good (normal) were combined to create three categories for analysis (normal, moderate difficulties, poor hearing).^10^, ^20^

#### Objective Hearing Test

A hearing screening device (HearCheck Screener, Siemens, Germany) was used to obtain objective hearing scores for participants at Wave 7 (2014/15). This device has been validated and was used in the Health Survey for England in 2014.^10^ Hearing loss was measured according to decibel hearing level (dbHL), which is the level in decibels needed for a person to hear a sound at a certain frequency at least half the time.^10^ The test involves presentation of six increasing volumes of sounds at different frequency levels; participants indicate which tones they can hear.^10^, ^21^ Both ears were tested in a quiet environment, and hearing aids were removed before the test.^10^ The test was not conducted with people who had a cochlear implant or ear infection.^10^ Individuals were classified as having hearing loss if they could hear only mid‐frequency sounds at 20 dbHL and high‐frequency sounds at 35 dbHL. Responses were originally categorized into four groups (good hearing, moderate loss, severe loss, profound loss). For the current study, the lower two (severe and profound loss) were combined, resulting in three groups (normal, moderate difficulties, poor hearing).

#### Other Independent Variables

Age was classified into four categories (50–59, 60–69, 60–79, ≥80). Economic status was defined using quintiles of non‐pension wealth (1 = low, 5 = high) as calculated by the Institute for Fiscal Studies.^13^ Participants' highest educational qualifications were divided into three groups (no formal qualification, intermediate education, higher education). Ethnicity was divided into white and non‐white. Smoking was categorized into three groups (never smoker, ex‐smoker, current smoker). The following variables were binary: use of hearing aid, diabetes mellitus, hypertension, history of stroke.^22^

### Statistical Analysis

The sociodemographic and clinical risk profiles were summarized according to self‐reported (Waves 2–7, 2004/05–2014/15) and objective (Wave 7, 2014/15) hearing categories. Chi‐square tests were performed to ascertain whether there were significant differences in the distribution of sociodemographic and clinical categories between hearing groups. For the cross‐sectional analyses of self‐reported and objective hearing impairment, odds ratios and 95% confidence intervals of diagnosed dementia at Wave 7 (2014/15) were calculated, with normal hearing as the reference group. Separate analyses were conducted on self‐reported and objective hearing impairment. On the basis of the existing literature, it was decided *a priori* that age, sex, ethnicity, wealth, education, and hearing aid use were possible confounders.^7^, ^11^, ^23^ The following cardiovascular risk factors were also adjusted for: smoking status, diabetes mellitus, hypertension, and stroke. A forward stepwise approach was used, and likelihood ratio tests and the Akaike Information Criterion were used to select the model of best fit.^24^, ^25^

Cox proportional hazards regression was used to model the association between self‐reported hearing (Wave 2, 2004/05) and cumulative diagnosed dementia (July 2005 to June 2015). The Health Survey for England has calculated cross‐sectional and longitudinal weighting to adjust for non‐response bias.^22^, ^26^ Cross‐sectional weights were derived for participants responding at each wave, and longitudinal weights were calculated using logistic regression models to estimate probability of non‐response using household‐ and individual‐level data collected in the previous wave.^22^ Significant differences were found between responders and non‐responders with regard to age, sex, region, highest education qualification, marital status, and self‐reported general health.^22^ Both weights were used to adjust for non‐response bias in the cross‐sectional (Wave 7, 2014/15) and longitudinal (Waves 2–7, 2004/05–2014/15) analyses.^22^

Individuals who had been diagnosed with dementia in Wave 2 (2004/05) were excluded. Time to dementia was measured in years from the beginning of Wave 2 (2004/05). Date of dementia diagnosis was used if known; if not known, the midpoint date between waves of data collection was used. Individuals who were known to have died or left the study were censored. Mortality data for ELSA were available up to February 2013. If an individual dropped out of the study between waves, the last interview date was used for the censor date. The Schoenfeld residual test was used to test the proportional hazards assumption of the models.^27^

### Sensitivity Analysis

To examine whether the self‐report and objective measures had independent effects on risk of dementia, both measures were included in one model. Individuals who wore hearing aids were excluded from the sensitivity analysis because the self‐report measure was based on hearing aid use, and the objective measure was not. Finally, to avoid misinterpreting the results of communication difficulties as a sign of cognitive impairment, a sensitivity analysis that omitted individuals who fit the IQCODE cut‐off criteria but did not have a physician diagnosis of dementia were included.

All data were analyzed using STATA version 14 (STATA Corp LP, College Station, TX).

## Results

### Cross‐Sectional Analyses

Ninety‐five percent (7,865/8,253) of participants in Wave 7 rated their hearing, with 23.1% (n = 1,771) reporting poor and 34.7% (n = 2,669) moderate hearing difficulties. Self‐reported hearing difficulties were associated with older age, male sex, lower wealth and education, hearing aid use, history of stroke, and diabetes or hypertension (Table 1).

**Table 1:** Participant Characteristics According to Self‐Reported Hearing Ability (Wave 7, 2014/15)

Characteristic	Total Cohort from Wave 7, n = 7,685	Self‐Reported Hearing			P‐Value*
		Poor, n = 1,771 (23.1%)	Moderate Difficulties, n = 2,669 (34.7%)	Normal, n = 3,242 (42.2%)	
	n (%)				
Dementia	193 (2.5)	86 (4.9)	67 (2.5)	40 (1.2)	<.001
Age					
50–59	937 (12.2)	131 (7.42)	287 (10.8)	519 (16.0)	<.001
60–69	3,139 (40.9)	571 (32.2)	1,054 (39.5)	1512 (46.6)	
70–79	2,414 (31.4)	610 (34.4)	888 (33.3)	914 (28.2)	
≥80	1,196 (15.5)	459 (25.9)	440 (16.5)	297 (9.2)	
Female	4,302 (55.9)	786 (43.4)	1,479 (55.4)	2,054 (63.4)	<.001
Wealth quartile					
1 (low)	1,292 (16.8)	377 (21.3)	448 (16.8)	466 (14.4)	<.001
2	1,384 (18.0)	368 (20.8)	479 (17.9)	535 (16.5)	
3	1,632 (20.8)	378 (21.3)	566 (21.2)	688 (21.2)	
4	1,694 (22.4)	339 (19.1)	595 (22.3)	760 (23.4)	
5 (high)	1,683 (22.0)	309 (17.5)	581 (21.8)	793 (24.5)	
Non‐white	261 (3.4)	51 (2.9)	105 (3.9)	105 (3.2)	.52
Education					
No qualifications	1,838 (23.9)	557 (31.5)	654 (24.5)	626 (19.3)	<.001
Intermediate	3,100 (40.3)	658 (37.2)	1,055 (39.5)	1,386 (42.8)	
Higher	2,747 (35.8)	556 (31.4)	960 (35.9)	1,230 (37.9)	
Hearing aid	1,041 (13.6)	557 (31.4)	354 (13.3)	130 (4.1)	<.001
Diabetes mellitus	1,070 (13.9)	329 (18.6)	352 (13.2)	389 (12.0)	<.001
Hypertension	3,836 (49.9)	1,006 (56.8)	1,362 (51.1)	1,006 (56.8)	<.001
Stroke	457 (5.9)	157 (8.9)	169 (6.3)	131 (4.1)	<.001
Current smoker	810 (10.5)	176 (9.9)	270 (10.1)	364 (11.2)	.17

Mean age 70.2 ± 9.5

Approximately 84% (6,902/8,253) of the participants had a hearing screening test in Wave 7 (TableS1). Fewer individuals were categorized into the poor objective hearing group (5%) than the poor self‐reported hearing group (23%), but a similar proportion (34% vs 35%) were in the moderate hearing group. Objective hearing difficulties were associated with a similar set of demographic and clinical factors as were self‐reported hearing difficulties (Table S1).

**Table 2:** Odds of Dementia According to Cross‐Sectional Self‐Reported Hearing and Objective Hearing Test (Wave 7, 2014/15)

Hearing Test	Model 1 (Unadjusted)	Model 2 (Adjusted)
	Odds Ratio (95% Confidence Interval) P‐Value	
Self‐reported hearing (reference normal)		
Moderate	2.06 (1.39–3.06) <.001	1.58 (1.05–2.37) .03
Poor	4.08 (2.79–5.97) <.001	2.62 (1.74–3.93) <.001
Age (reference 50–59)		
60–69	1.65 (0.57–4.79) .36	1.70 (0.58–4.97) .33
70–79	5.34 (1.93–14.8) .001	4.95 (1.77–13.8) .002
≥80	24.6 (9.03–66.9) <.001	21.2 (7.67–58.7) <.001
Female	1.12 (0.84–1.50) .43	0.93 (0.66–1.26) .66
Wealth quartile (reference 1)		
2	0.69 (0.47–1.02) .07	0.77 (0.51–1.17) .22
3	0.56 (0.37–0.83) .001	0.71 (0.46–1.08) .11
4	0.35 (0.22–0.54) .001	0.53 (0.32–0.87) .01
5	0.22 (0.13–0.38) .001	0.43 (0.24–0.77) .005
Education (reference no qualifications)		
Intermediate	0.33 (0.23–0.46) <.001	0.54 (0.38–0.78) .001
Higher	0.29 (0.20–0.42) <.001	0.71 (0.47–1.08) .11
Hearing aid	0.65 (0.40–1.05) .08	0.24 (0.14–0.39) .04
Hypertension	2.59 (1.89–3.56) <.001	1.56 (1.11–2.17) .01
Stroke	7.38 (5.33–10.2) <.001	4.04 (2.83–5.76) <.001
Objective hearing (reference normal)		
Moderate	2.41 (1.44–4.03) .001	1.62 (0.93–2.84) .09
Poor	7.54 (4.01–14.2) <.001	4.39 (1.94–9.91) <.001
Age (reference 50–59)		
60–69	1.06 (0.35–3.22) .92	1.16 (0.38–3.56) .79
70–79	2.69 (0.94–7.69) .07	2.39 (0.81–7.07) .11
≥80	7.02 (2.47–19.9) <.001	4.55 (1.49–13.1) .008
Female	1.13 (0.71–1.79) .59	1.04 (0.64–1.68) .64
Wealth quartile (reference 1)		
2	0.37 (0.18–0.75) .06	0.46 (0.23–0.96) .23
3	0.51 (0.28–0.94) .03	0.64 (0.34–1.20) .34
4	0.32 (0.16–0.63) .001	0.41 (0.19–0.85) .19
5	0.21 (0.09–0.47) <.001	0.33 (0.14–0.78) .14
Education (reference no qualifications)		
Intermediate	0.38 (0.21–0.66) .001	0.63 (0.35–1.10) .10
Higher	0.43 (0.25–0.73) .002	0.99 (0.53–1.86) .99
Hearing aid	0.68 (0.42–1.05) .08	0.24 (0.24–0.99) .046
Hypertension	1.71 (1.07–2.72) .02	1.06 (0.65–1.72) .82
Stroke	4.32 (2.39–7.79) <.001	2.48 (1.34–4.61) .004

Dementia was associated with worse self‐reported and objective hearing (Table 2). After adjustment for confounders, participants in the moderate and poor self‐reported hearing groups were 1.6 (95% CI = 1.05–2.37) and 2.6 (95% CI = 1.74–3.93) times more likely to have a dementia diagnosis as those with normal hearing. Similarly, those in the moderate and poor hearing groups for the hearing screening test were 1.6 (95% CI = 0.93–2.84) and 4.4 (95% CI = 1.94–9.91) times as likely to have a diagnosis of dementia. Older age, hypertension, and previous stroke were risk factors for dementia diagnosis, whereas greater wealth, intermediate and higher education, and using a hearing aid seemed to have protective effects (Table 2).

### Longitudinal Analyses

Of the 8,780 core members in Wave 2, 22% (n = 1,933) reported poor hearing, and 31.6% (n = 2,774) moderate hearing difficulties. Self‐reported hearing difficulty was associated with older age, male sex, lower wealth and education, hearing aid use, hypertension, and history of stroke (Table 3).

**Table 3:** Participant Characteristics According to Self‐Reported Hearing Ability (Wave 2–2004/05)

Characteristic	Total Cohort from Wave 2, n = 8,780	Self‐Reported Hearing			P‐Value
		Poor, n = 1,933 (22.0%)	Moderate Difficulties, n = 2,774 (31.6%)	Normal, n = 4,073 (46.4%)	
	N (%)				
Age					
50–59	2,597 (29.6)	371 (19.2)	772 (27.8)	1,454 (35.7)	<.001
60–69	2,874 (32.7)	537 (27.8)	941 (33.9)	1,396 (34.3)	
70–79	2,188 (24.9)	599 (31.0)	694 (25.0)	895 (22.0)	
≥80	1,121 (12.8)	426 (22.0)	367 (13.2)	328 (8.10)	
Female	4,831 (55.0)	848 (43.9	1,468 (52.9)	2,515 (61.7)	<.001
Wealth quartile					
1 (low)	1,583 (18.3)	445 (23.0)	475 (17.1)	663 (16.3)	<.001
2	1,724 (19.9)	459 (23.7)	523 (18.9)	742 (18.2)	
3	1,741 (20.1)	372 (19.2)	557 (20.1)	812 (19.9)	
4	1,773 (20.5)	334 (17.3)	579 (20.9)	860 (21.1)	
5 (high)	1,840 (21.2)	303 (15.7)	605 (21.8)	932 (22.9)	
Non‐white	206 (2.2)	52 2.7)	55 (2.0)	99 (2.4)	.26
Education					
No qualifications	3,487 (39.6)	912 (47.2)	1,080 (38.9)	1,476 (36.2)	<.001
Intermediate	3,219 (36.6)	647 (33.5)	997 (35.9)	1,565 (38.4)	
Higher	2,100 (23.9)	371 (19.2)	693 (25.0)	1,029 (25.3)	
Hearing aid	561 (6.5)	385 (19.9)	143 (5.2)	41 (1.0)	<.001
Diabetes mellitus	248 (2.8)	72 (3.7)	76 (2.7)	100 (2.5)	.02
Hypertension	1,079 (12.3)	273 (14.1)	362 (13.0)	444 (10.9)	.001
Stroke	142 (1.6)	55 (2.8)	43 (1.6)	44 (1.1)	<.001
Current smoker	1,329 (15.1)	304 (15.7)	412 (14.9)	613 (15.1)	.69

There were 269 incident cases of diagnosed dementia between Wave 2 and the end of Wave 7 (June 2015). During the mean follow‐up of 11 years, individuals in the moderate (hazard ratio (HR) = 1.39, 95% CI = 1.01–1.92) and poor (HR = 1.57, 95% CI = 1.12–2.02) hearing groups were at greater risk of developing dementia than the normal hearing group. Older age and diabetes mellitus also emerged as significant independent risk factors (Table 4).

**Table 4:** Hazard Ratio of Self‐Reported Hearing at Wave 2 (2004/05) and Cumulative Dementia (Waves 3–7, 2006/07–2014/15)

Characteristic	No Dementia, n = 8,382	Dementia, n = 269	Hazard Ratio (95% Confidence Interval) P‐Value
	n (%)		
Self‐reported hearing			
Normal	3,921 (46.8)	85 (31.6)	1
Moderate	2,645 (31.6)	90 (33.5)	1.39 (1.01–1.92) .04
Poor	1,816 (21.7)	94 (34.9)	1.57 (1.12–2.02) .009
Age			
50–59	2,520 (30.1)	19 (7.1)	1
60–69	2,795 (33.4)	45 (16.7)	2.06 (1.19–3.66) .01
70–79	2,062 (24.6)	101 (37.6)	6.44 (3.86–10.7) <.001
≥80	1,005 (12.0)	108 (40.2)	18.3 (10.6–31.5) <.001
Sex			
Male	3,791 (45.2)	103 (38.3)	1
Female	4,591 (54.8)	172 (63.9)	1.09 (0.84–1.41) .52
Wealth quartile			
1	1,525 (18.2)	55 (20.5)	1
2	1,650 (19.7)	69 (25.7)	1.34 (0.97–2.11) .07
3	1,688 (20.2)	53 (19.7)	1.06 (0.75–1.71) .57
4	1,725 (20.6)	48 (17.8)	1.08 (0.71–1.71) .66
5	1,794 (21.4)	44 (16.4)	1.04 (0.67–1.87) .65
Education			
No qualifications	3,295 (39.3)	142 (52.8)	1
Intermediate	3,082 (36.7)	79 (29.4)	0.78 (0.56–1.03) .07
Higher	2,005 (23.9)	48 (17.8)	0.79 (0.53–1.16) .22
Hearing aid			
No	7,850 (93.7)	238 (88.5)	1
Yes	532 (6.3)	31 (11.5)	0.99 (0.61–1.42) .74
Hypertension			
No	7,354 (87.7)	228 (84.8)	1
Yes	1,028 (12.3)	41 (15.2)	1.01 (0.68–1.52) .94
Stroke			
No	8,249 (98.4)	262 (97.4)	1
Yes	133 (1.6)	7 (2.6)	0.82 (0.33–1.57) .41
Diabetes mellitus			
No	8,150 (97.3)	253 (94.1)	1
Yes	230 (2.7)	16 (5.9)	2.39 (1.35–4.57) .003

### Sensitivity Analysis

There was fair agreement between the objective and self‐reported hearing measures (*κ* = 0.262, 95% CI = 0.257–0.269). The association between objective hearing and physician‐diagnosed dementia remained significant for the poor hearing group when including self‐reported hearing in the model. In contrast, the association between self‐reported hearing difficulties weakened and became non‐significant. The associations did not change when individuals who used a hearing aid were excluded. When individuals with an IQCODE greater than 3.5 only were omitted from the model, the odds ratios were smaller but remained significant.

## Discussion

The study found that moderate and poor objective and self‐reported hearing were cross‐sectionally associated with physician‐diagnosed dementia in a representative sample of English older adults (mean age 70 ± 9.5). Longitudinal analysis over an 11‐year period showed that the incidence was 39% higher in individuals with moderate self‐reported hearing and 57% higher in those with poor self‐reported hearing than in those with normal hearing after adjusting for multiple covariates.

### Comparison with Other Studies

The Health and Retirement Study, which has a profile similar to that of ELSA, also examined self‐reported hearing and dementia cross‐sectionally and found that 44% of participants who reported fair to poor hearing had probable dementia.^28^ The Health and Retirement Study analysis focused on the last 2 years of life rather than a longer period, and their definition of probable dementia was based on an algorithmic analysis of cognitive function rather than physician diagnosis.^28^, ^29^

The current study built on previous longitudinal studies conducted in the United States and Wales that found that individuals with moderate and severe hearing loss were at greater risk of developing dementia.^6^, ^7^, ^12^, ^22^ Six hundred thirty‐nine adults aged 65 and older from the Baltimore Longitudinal Study of Aging were prospectively analysed over 11.9 years.^7^ It was found not only that objective hearing loss was independently associated with incident dementia, but also that risk increased log‐linearly with severity of hearing loss (mild hearing loss: HR = 1.89, 95% CI = 1.00–3.58; moderate hearing loss: HR = 3.00, 95% CI = 1.43–6.30; severe hearing loss, HR = 4.94, 95% CI = 1.09–22.40).^7^ The CI for the severe hearing loss category was large, probably because of the small number of cases in that category (n = 6). Another study tracked older adults (N = 1,889, aged 70–79) from the Health, Aging and Body Composition Study for 9 years and found that individuals with moderate to severe objective hearing impairment were more likely to develop dementia than those whose hearing was normal (HR = 1.55, 95% CI = 1.10–2.19). Older men from the Caerphilly Study in Wales (N = 1,057) were followed for 17 years, and an association was found between objective auditory threshold and dementia (OR = 2.67, 95% CI = 1.38–5.18). Unlike the other previous studies, they included only men in their analysis.^6^ Finally, the Cache County Study on Memory, Health, and Aging in the United States (N = 4,545) followed older adults (≥65) for 13 years and also found that hearing loss was an independent risk factor for developing dementia,^12^ although identification of hearing loss was questionable because it was based on interviewer ratings that were not a mandatory part of the assessment protocol.

The previous studies of objective hearing loss used a pure tone audiometry test, whereas the current study used self‐reported hearing measures in the longitudinal analyses.^6^, ^7^, ^11^ Nevertheless, a significant positive association was found between the self‐reported and objective test measure in Wave 7. In addition, pure tone audiometry and the hearing screening test used are comparable, with similar sensitivities (89% and 94%) and specificities (87% and 82%).^30^ When self‐reported and objective measures were entered into the same models competitively, associations between objective hearing loss and dementia were more robust.

### Strengths and Limitations

A unique strength of using ELSA is that it involves a large national sample of resident English men and women aged 50 and older. The dataset includes repeated measures of chronic conditions, so it was possible to capture cumulative physician‐diagnosed dementia cases and analyze time to dementia diagnosis. The dataset also had measures of self‐reported and objective hearing measures at Wave 7 that could be compared in the presence of other potential covariate measures.

There are also several limitations to these analyses. First, there were fewer individuals with dementia in this study than in population estimates,^31^, ^32^ primarily because dementia was identified on the basis of physician diagnosis; it is thought that only approximately half of people living with dementia have a formal diagnosis.^33^ Individuals with dementia based on an IQCODE score greater than 3.5 were therefore also included.^15^, ^29^, ^34^ Attrition bias is also relevant,^27^ although this was allowed for by using probability weights for non‐responders.^22^, ^26^

Second, only self‐reported hearing measures were available in ELSA for longitudinal analysis; objective measures would be desirable. Comparison of subjective and objective measures of hearing loss was also challenging, because only self‐reported hearing was based on hearing aid use, although the two measures had a significant positive association. In addition, use of a hearing aid was included as a confounder in the analysis.

Finally, categorizations of subjective and objective hearing measures do not match directly because they are based on rather different criteria.

### Possible Causal Mechanisms

Although longitudinal associations between self‐reported hearing loss and future dementia were identified, it is not certain that the associations are causal. Major confounders such as age, education, and cardiovascular risk factors were adjusted for statistically, but there could be residual confounding by unmeasured variables. In addition, dementia develops over many years, so pathological processes may have been initiated even before baseline measures of hearing. Nevertheless, there are three possible mechanisms by which hearing loss could cause dementia; cognitive burden, changes in brain structure and function, and social isolation. First, cognitive burden or the “effortfulness hypothesis” has been demonstrated in older adults (66–81).^34^, ^35^ Although the numbers of older adults with hearing impairment were small (n = 12, n = 24), findings suggested that older individuals with hearing loss had poorer recall and secondary task performance.^35^, ^36^ Sound signals become more distorted in individuals with hearing loss, especially in the high‐frequency range, leading to greater effort in perceiving sound.^35^, ^36^ The extra cognitive load on individuals with hearing loss could be at the expense of encoding and processing speech into memory.^35^, ^36^

Second, lack of sensory input and difficulty processing may lead to changes in brain structure and function.^6^, ^7^, ^8^ Evidence from cross‐sectional studies suggests that hearing impairment is associated with a reduction in the cortical volume of the primary auditory cortex in the temporal lobe and variable white matter fibers.^37^ Neuroimaging data from participants enrolled in the neuroimaging substudy of the Baltimore Longitudinal Study of Aging strengthened the evidence, showing that hearing impairment in older adults was associated with a greater rate of decline in whole brain volume, particularly in the right temporal lobe, which is responsible for processing of speech.^38^ The majority of individuals (n = 126, aged 58–86) with hearing impairment were in the mild hearing category.

Finally, lack of interaction and intellectual stimulation has been associated with dementia in prospective studies.^38^, ^39^ Social gatherings may be more challenging for individuals with hearing impairment because they use more cognitive resources to process speech, which may increase withdrawal from social activities. Swedish older adults were examined prospectively (n = 776, aged ≥75), and the results suggested that individuals who participated less in social, mental, or physical activity had a greater risk of developing dementia.^39^ Furthermore, cross‐sectional and prospective studies have shown an independent association between hearing loss and social isolation.^40^ Social isolation may therefore be a mediator on the causal pathway of dementia through which hearing impairment acts.^40^

### Clinical Implications

Is hearing loss an indicator of early stages of dementia and a preventable risk factor, or is dementia an indicator of hearing loss? There are opposing arguments regarding the direction of the association between hearing and dementia, but either pathway could have major public health implications.

In the United Kingdom, approximately 4 million people with hearing impairment delay seeking medical help.^41^ Unlike eye tests, individuals seem to be reluctant to have hearing tests, possibly because of the stigma associated with hearing loss, which could be reduced by using a screening program.^41^ One study of hearing screening for older adults showed a positive benefit to cost ratio.^42^ Consequently, the Department of Health has developed an action plan on hearing that includes awareness, early detection, and treatment for hearing loss.^43^

Hearing aids could help with treatment of hearing loss and possibly decrease social isolation, although they need to be acceptable and effective. The quality of hearing aids seems to have improved, and 70% of older adults reported being fairly satisfied with their hearing aids in the Health Survey for England.^10^ Individuals who were tested at a younger age benefitted more from their hearing aids because they had more time to adapt.^41^ Combined with detection and treatment, hearing loss could also be an early indicator for cognitive decline and dementia.

## Conclusion

This study supports previous literature that has found that older adults with hearing loss have a higher rate of developing dementia. It also found that hearing aid use had a protective effect in cross‐sectional analysis. The findings suggest that treatment of hearing loss with hearing aids could help delay the onset of dementia. The public health implications are substantial because more than 3 million U.K. adults aged 50 and older have hearing loss. Further studies are needed to confirm the possible biological and social mechanisms involved, and a large prospective study is needed to examine treatment of hearing loss.

## Supporting information


**Table S1.** Descriptive statistics of dementia and objective hearing test
