# Supplementary material for: Hearing Impairment and Incident Dementia: Findings from the English Longitudinal Study of Ageing
Source: J Am Geriatr Soc. 2017 Jul 22;65(9):2074–81. doi: 10.1111/jgs.14986 (PMC5637915; doi:10.1111/jgs.14986)
Supplement: Supplementary file 1 — Table S1. Descriptive statistics of dementia and objective hearing test [file JGS-65-2074-s001.docx]

**Supplementary Table S1:** Descriptive statistics of dementia and objective hearing test

|  | | | **Objective hearing screening test (Wave 7)** | | | | | | | | | | |
| --- | --- | --- | --- | --- | --- | --- | --- | --- | --- | --- | --- | --- | --- |
|  | **Total cohort from wave 7**  **(n= 6,902)** | | | | **Poor**  **(n=359, 5.2%)** | | **Moderate difficulties**  **(n=2,314, 33.5%)** | | **Normal**  **(n=4,229, 61.3%)** | | | **p-value** | |
|  | **N** | **%** | | **N** | | **%** | **N** | **%** | | **N** | **%** | |  |
| **Dementia** | 76 | 1.12 | | 16 | | 4.40 | 34 | 1.51 | | 26 | 0.61 | | <0.001 |
| **Age groups (years)**  50-59  60-69  70-79  80+ | 865  2,865  2,191  981 | 12.5  41.5  31.7  14.2 | | 11  38  122  188 | | 3.12  10.6  34.0  52.4 | 145  690  907  572 | 6.30  29.8  39.2  24.7 | | 709  2,137  1,162  221 | 16.8  50.5  27.5  5.21 | | 0.001 |
| **Gender**  Females | 3,880 | 56.2 | | 184 | | 51.3 | 1,198 | 52.0 | | 2,498 | 59.1 | | <0.001 |
| **Wealth**  1  2  3  4  5 | 1,128  1,218  1,450  1,555  1,551 | 16.3  17.7  21.0  22.5  22.5 | | 109  69  86  58  37 | | 30.4  19.2  24.0  16.2  10.3 | 447  440  465  536  426 | 19.3  19.0  20.1  23.2  18.4 | | 572  709  899  961  1,088 | 13.5  16.8  21.3  22.7  25.7 | | 0.001 |
| **Ethnicity**  Non-white | 218 | 3.21 | | 4 | | 1.10 | 79 | 3.42 | | 139 | 3.30 | | 0.18 |
| **Education**  No qualifications  Intermediate  Higher | 1,574  2,814  2,514 | 22.8  40.8  36.4 | | 160  118  81 | | 44.6  32.9  22.6 | 683  919  712 | 29.5  39.7  30.8 | | 731  1,777  1,721 | 17.3  42.0  40.7 | | 0.001 |
| **Hearing aid**  Yes | 896 | 13.0 | | 260 | | 72.4 | 536 | 23.2 | | 100 | 2.41 | | <0.001 |
| **Diabetes**  Yes | 931 | 13.5 | | 64 | | 17.8 | 378 | 16.3 | | 489 | 11.6 | | <0.001 |
| **Hypertension**  Yes | 3,368 | 48.8 | | 223 | | 62.1 | 1,270 | 54.9 | | 1,875 | 44.3 | | <0.001 |
| **Stroke**  Yes | 353 | 5.12 | | 42 | | 8.91 | 160 | 6.30 | | 151 | 4.12 | | <0.001 |
| **Smoking status**  Current | 712 | 10.3 | | 31 | | 8.62 | 223 | 9.61 | | 458 | 10.8 | | 0.17 |
